# Design, Synthesis, Computational, and Preclinical Evaluation of ^nat^Ti/^45^Ti-Labeled Urea-Based Glutamate PSMA Ligand

**DOI:** 10.3390/molecules25051104

**Published:** 2020-03-02

**Authors:** Kristina Søborg Pedersen, Christina Baun, Karin Michaelsen Nielsen, Helge Thisgaard, Andreas Ingemann Jensen, Fedor Zhuravlev

**Affiliations:** 1 Department of Health Technology, Technical University of Denmark, Frederiksborgvej 399, Building 202, 4000 Roskilde, Denmark; krped@dtu.dk (K.S.P.); kamnie@dtu.dk (K.M.N.); atije@dtu.dk (A.I.J.); 2 Department of Clinical Research, University of Southern Denmark, Sønder Boulevard 29, DK-5000 Odense, Denmark; Christina.Baun@rsyd.dk (C.B.); Helge.Thisgaard@rsyd.dk (H.T.); 3 Department of Nuclear Medicine, Odense University Hospital, DK-5000 Odense, Denmark

**Keywords:** titanium-45, PSMA, PET

## Abstract

Despite promising anti-cancer properties in vitro, all titanium-based pharmaceuticals have failed in vivo. Likewise, no target-specific positron emission tomography (PET) tracer based on the radionuclide ^45^Ti has been developed, notwithstanding its excellent PET imaging properties. In this contribution, we present liquid–liquid extraction (LLE) in flow-based recovery and the purification of ^45^Ti, computer-aided design, and the synthesis of a salan-^nat^Ti/^45^Ti-chelidamic acid (CA)-prostate-specific membrane antigen (PSMA) ligand containing the Glu-urea-Lys pharmacophore. The compound showed compromised serum stability, however, no visible PET signal from the PC3+ tumor was seen, while the ex vivo biodistribution measured the tumor accumulation at 1.1% ID/g. The in vivo instability was rationalized in terms of competitive citrate binding followed by Fe(III) transchelation. The strategy to improve the in vivo stability by implementing a unimolecular ligand design is presented.

## 1. Introduction

Since its inception in 1975 [1], PET has become one of the most useful and rapidly developing diagnostic modalities in the field of oncology, cardiology, and neurology [2]. Following its clinical success and rapid development, the global market for PET radiopharmaceuticals grew from $1.5 billion in 2014 to an estimated $2.3 billion in 2019 and is expected to reach $2.8 billion by 2022 [3]. PET radiopharmaceuticals (tracers) provide functional imaging of disease by precise molecular targeting of the affected tissue. To match the time-scale of the biological process under investigation, PET tracers utilize a broad spectrum of positron-emitting radionuclides. Among these, radiometals are particularly attractive as they combine widely varying half-lives with an ease of radiolabeling via kit formulations [4]. While the radionuclides ^68^Ga and ^82^Rb still dominate the traditional clinical setting, the use of ^64^Cu [5] and ^89^Zr [6] is on the rise in university clinics and clinical trials, and a plethora of PET tracers based on more unconventional PET radiometals such as ^44^Sc, ^45^Ti, ^55^Co, and ^86^Y are in development [7]. The radionuclide ^45^Ti occupies a special place among unconventional PET radiometals, featuring 85% β^+^ decay, negligible secondary radiation, and low β^+^ endpoint energy (1.04 MeV), which translates into high spatial resolution as evidenced by sharp Derenzo phantom images [8,9,10]. With its 3.1 h half-life, ^45^Ti is well-suited for the radiolabeling of small molecules, peptides, and antibody fragments. The three hours half-life also allows for regional distribution and longer transport distances compared to ^68^Ga, and makes it possible to perform delayed PET imaging. The case in point is PSMA-based PET diagnostics, where imaging 3 h post-injection (p.i.) is considered to produce the highest contrast allowing the discovery of additional lesions [11]. The production of ^45^Ti via the ^nat^Sc(p,n)^45^Ti reaction is convenient and high-yielding, allowing for 4.6 GBq of activity after one hour of irradiation of Sc foil using medical cyclotron (11.8 MeV protons, 20 µA) [10]. The naturally monoisotopic Sc is inexpensive and readily available.

The imaging and production advantages of ^45^Ti are offset by the chemical challenges of recovering the radionuclide from the irradiated Sc matrix. The high oxophilicity of Ti^4+^ (Ө = 1.0) [12] and propensity for hydrolysis require strong acidities of the digestion solution ([H^+^] > 8 M) to avoid the formation of titanyl species [13]. The separation of ^45^Ti from Sc has been previously performed using various resins. The cation exchange resin AG 50W-X8 allowed for as much as 92.3% recovery of the ^45^Ti activity from the 6 M HCl. However, the IR analysis indicated the predominance of the titanyl [^45^Ti]TiOCl_2_ species in the dried-down sample [8]. A hydroxamic acid resin with oxalic acid elution was also used, but the yields were modest (42%–56%) [10,14]. Higher recovery yields (93%) could be obtained on a HypoGel 1,3-diol resin in organic solvents, but the procedure required on-resin chelation chemistry. Recently, we reported the efficient LLE of ^45^Ti from Sc-containing 12 M HCl using a guaiacol/anisole mixture [15]. The extraction is very fast and can be performed manually at low radioactivity levels. By using a membrane-based separator with integrated pressure control, we achieved a reliable and robust extraction with clean phase separation at high flow rates and GBq levels of activity. However, the purification of the product from the guaiacol/anisole mixture could be tedious and require preparative HPLC.

The efficient and stable chelation of titanium also presents a challenge. The cyclen family of popular chelators, such as 1,4,7,10-Tetraazacyclododecane-1,4,7,10-tetraacetic acid (DOTA) and its derivatives, has not demonstrated any utility for chelating Ti^4+^. Although the tetradentate diamino bis-phenolato family of ligands (salan) do allow for the fast and quantitative chelation of Ti^4+^, the resulting cytotoxic Ti(salan) complexes are prone to hydrolysis [16]. Supplementing salan with dipicolinic acid (dipic) leads to a heptacoordinate geometry around titanium and to a significant boost in the hydrolytic stability of the resulting salan-Ti-dipic, which retains its cytotoxicity [17]. Disappointingly, the PET radio-isotopomer [^45^Ti]salan-Ti-dipic showed negligible accumulation in the tumor tissue and fast hepatobiliary excretion [18]. The difficulties in recovery and purification and the absence of an in-vivo stable bifunctional chelator for ^45^Ti highlights the challenges of bringing this otherwise promising PET radionuclide into clinics and explains why no target-specific ^45^Ti PET tracers have been reported so far.

In this study, we aimed at overcoming the ^45^Ti purification challenge by the recovery of radiotitanium using LLE. We attempted to develop a hydrolytically stable, target-specific ^45^Ti-based tracer by modifying the previously reported salan-dipic scaffold into a bifunctional chelating moiety facilitating the labeling of a targeting vector. Given the growing importance of PSMA as a molecular targeting vector, we sought to design the first ^45^Ti-containing PSMA PET tracer by accessing its molecular compatibility with the glutamate carboxypeptidase II (GCPII, also known as PSMA) active site in silico, synthesize the compound, and test its performance in vivo using ^45^Ti PET and ex vivo organ biodistribution.

## 2. Results

### 2.1. Flexible Docking of Salan-Ti-CA-PSMA into the Active Site of GCPII

**Scheme 1:**
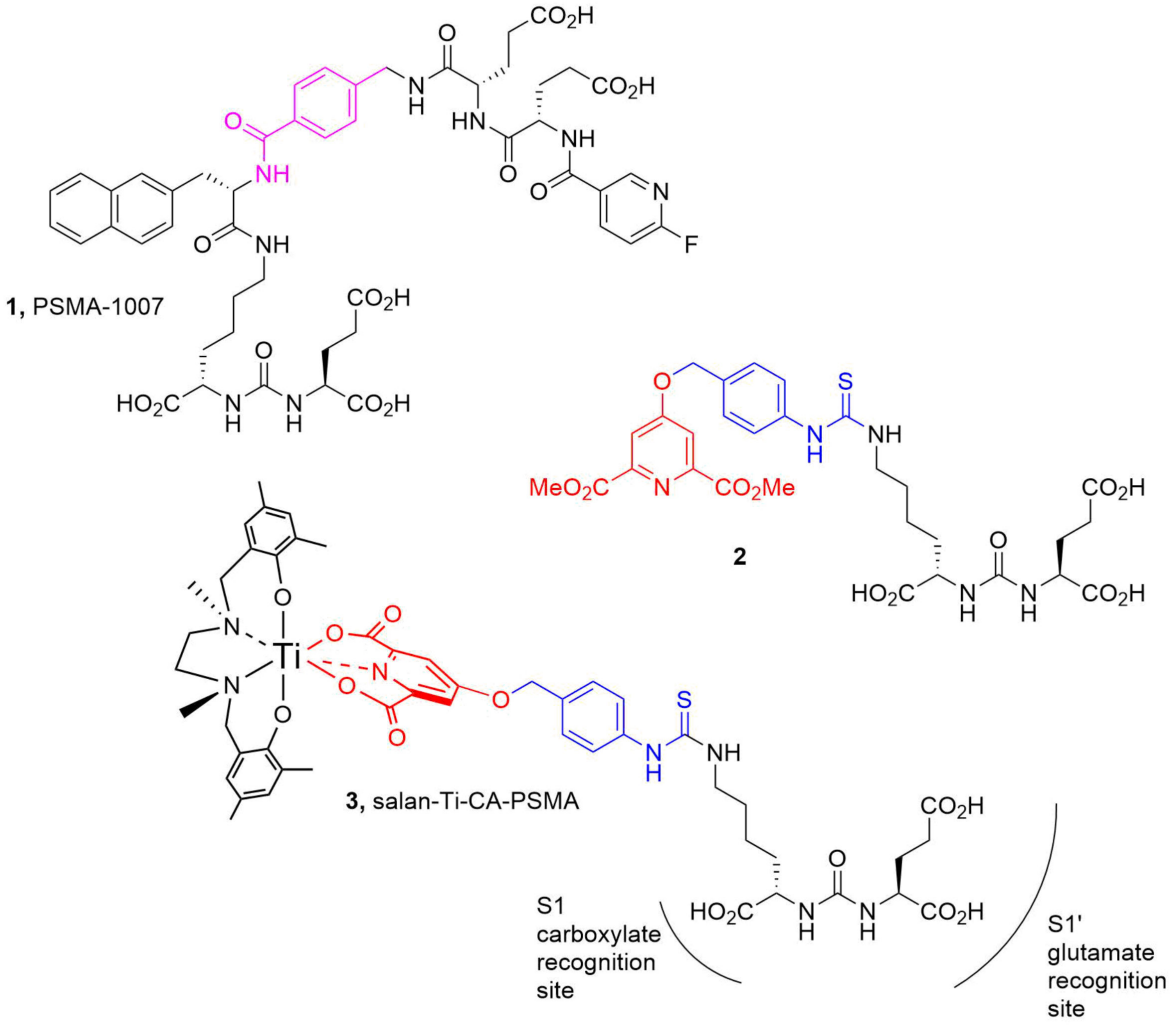
The structures used in the flexible docking studies (protein: PDB ID: 5O5T). The linker fragments are colored blue and pink; the chelidamic acid (CA) residue is red. PDB: Protein Data Bank.

The active site of the human recombinant GCPII was modeled using the crystal structure from the Protein Data Bank (PDB ID: 5O5T, 1.43Å resolution) containing PSMA-1007 (**1**) as a cognate ligand. Since the OPLS3 force field lacked the necessary parameters for Ti, the structure (**2**), containing the Glu-urea-Lys pharmacophore, the linker (Scheme 1, blue), and the chelidamic acid (CA) residue (Scheme 1, red) were used for molecular docking. Then, the docked structure was further functionalized by attaching the salan-Ti-CA moiety at the benzylic ester linkage forming compound (**3**), which is henceforth referred to as salan-Ti-CA-PSMA (**3**).

**Figure 1:**
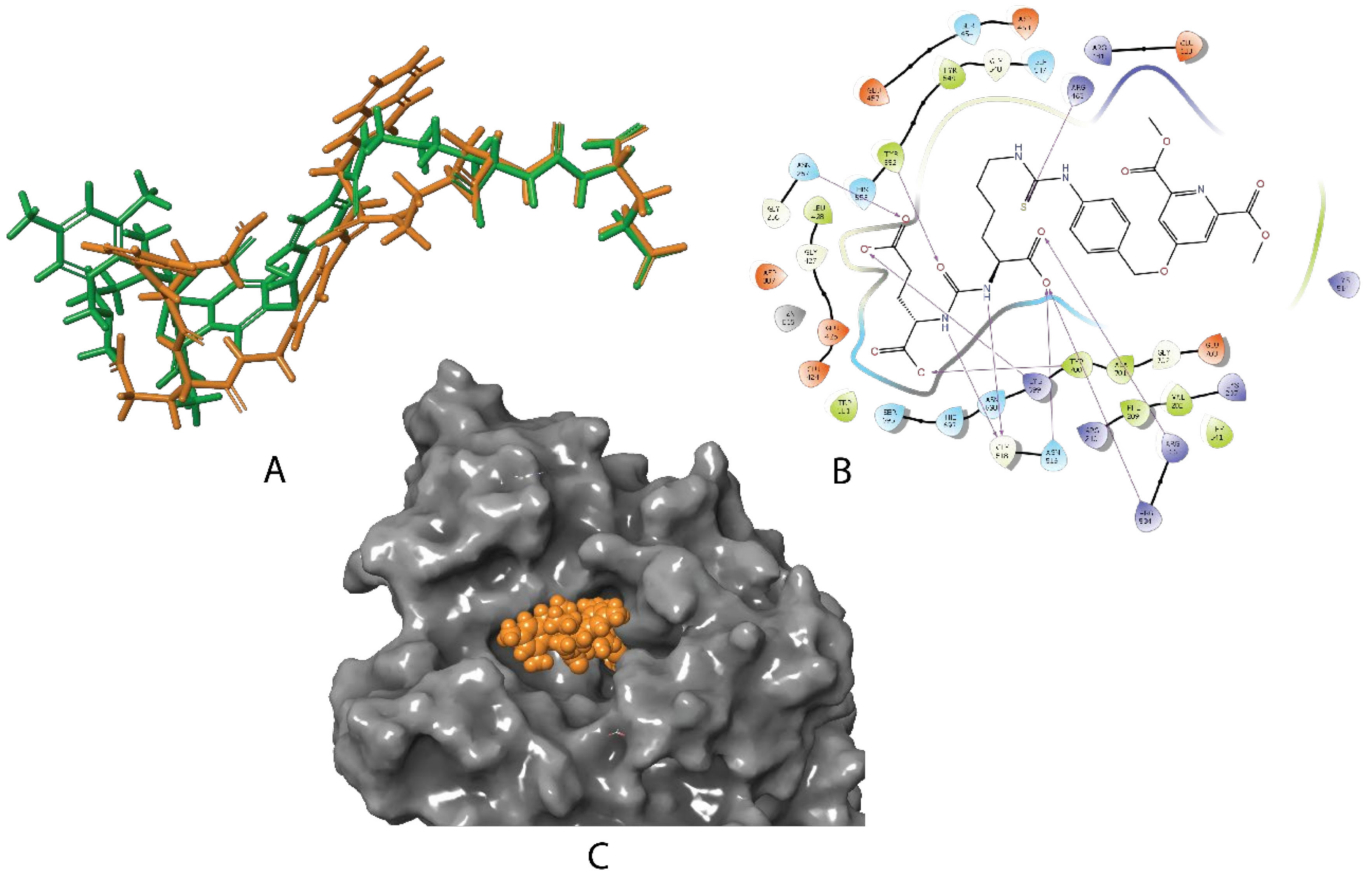
(**A**): Overlay of the best poses for **2** (green) with the cognate ligand **1** (PSMA-1007) (orange) as it is co-crystallized with glutamate carboxypeptidase II (GCPII) in the active site of PSMA (PDB ID: 5O5T); (**B**): the corresponding binding interaction of **2** with the active site schematically depicted in 2D; (**C**): the molecular surface of the protein (gray) and the best pose for **3** represented as CPK (Corey-Pauling-Koltun) model (orange).

The docking studies revealed striking similarities between the mode of active site binding in (**3**) and in the co-crystallized (**1**), PSMA-1007 (Figure 1). The carbonyl group of the urea is anchored to Zn^2+^ (815), in both structures, while the carboxylate groups of the P1′ formed hydrogen bonds to Arg 210, Asn 257, Tyr 552, Tyr 700, and Lys 699 of the S1′glutamate recognition site of the protein. The interaction with Arg 463 locks in place the thiourea carbonyl in (**3**) and the corresponding amide carbonyl in (**1**) forcing the benzylic groups to become coplanar and occupy almost the same space in the active site. The significant difference in binding between (**1**) and (**3**) is in the positioning of the terminal residues. While the fluoropyridine ring of (**1**) is forced into a small pocket formed by Trp 541, Arg 511, and Arg 463, the bulky salan-Ti-CA chelate is protruding into a much larger opening shaped by Arg 511, Tyr 709, Arg 181, and Lys 545. The structure (**3**) also lacks the stabilizing interaction of the naphthalene residue with the hydrophobic accessory pocket of the protein, which can affect the overall binding.

### 2.2. Synthesis of CA-PSMA and Nonradioactive Salan-Ti-CA-PSMA

**Scheme 2:**
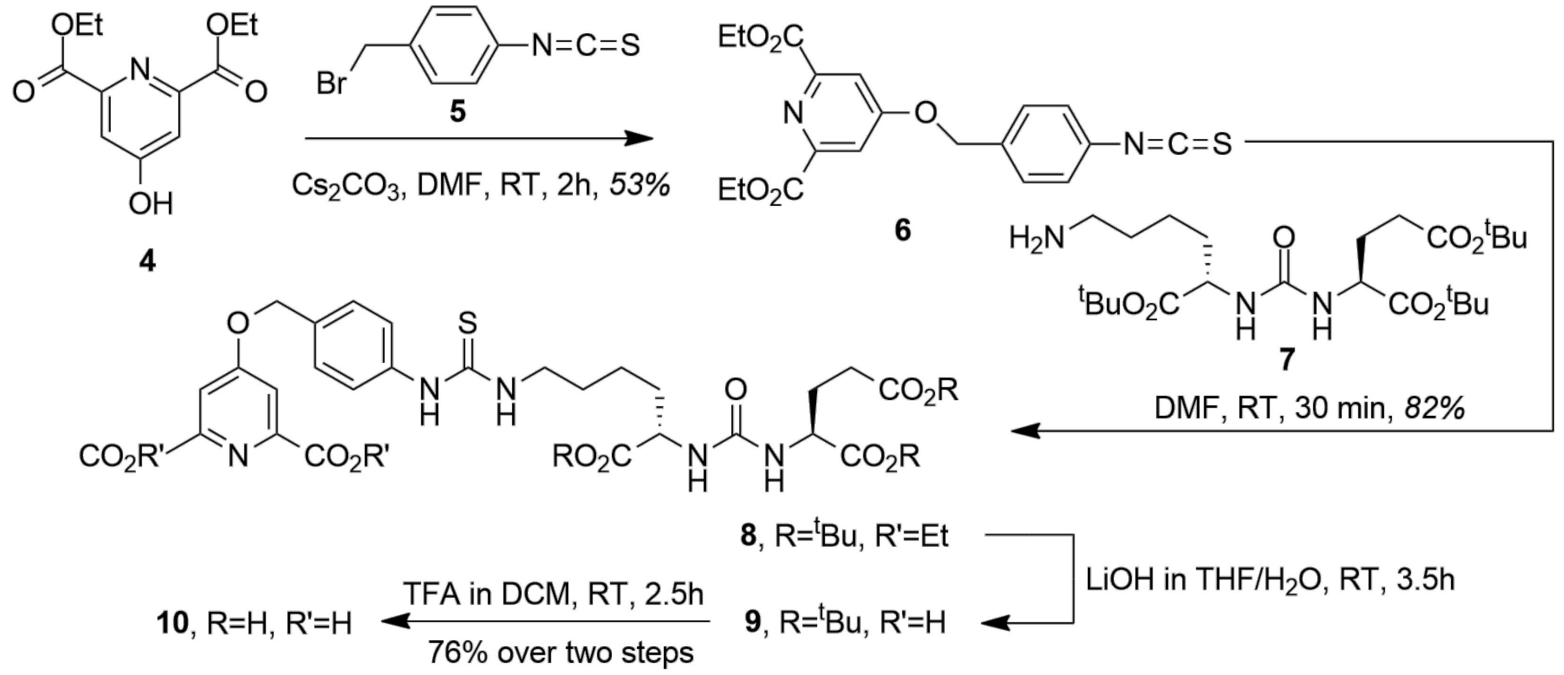
Synthesis of CA-PSMA (**10**).

The synthesis of the chelating pharmacophore CA-PSMA (**10**) is presented in Scheme 2. The room temperature *O*-alkylation of the chelidamic acid diethyl ether (**4**) [19] with benzylic bromide (**5**) [20] yielded the corresponding isothiocyanate (**6**), which was coupled with (**7**) and synthesized as described by Maresca et al. [21] to yield (**8**). The ethyl esters were saponified with LiOH in tetrahydrofuran (THF)/water, giving (**9**) followed by removal of the *tert*-butyl groups with trifluoroacetic acid (TFA) in dichloromethane (DCM) to give CA-PSMA (**10**). Overall, the synthesis of CA-PSMA (**10**) was performed in eight steps with the longest linear sequence of six steps and the combined yield of 28%. The synthesis duration and relatively low overall yield challenge the potential translation into routine production, and further optimizations of the synthesis are necessary to facilitate an efficient upscaling.

**Scheme 3:**
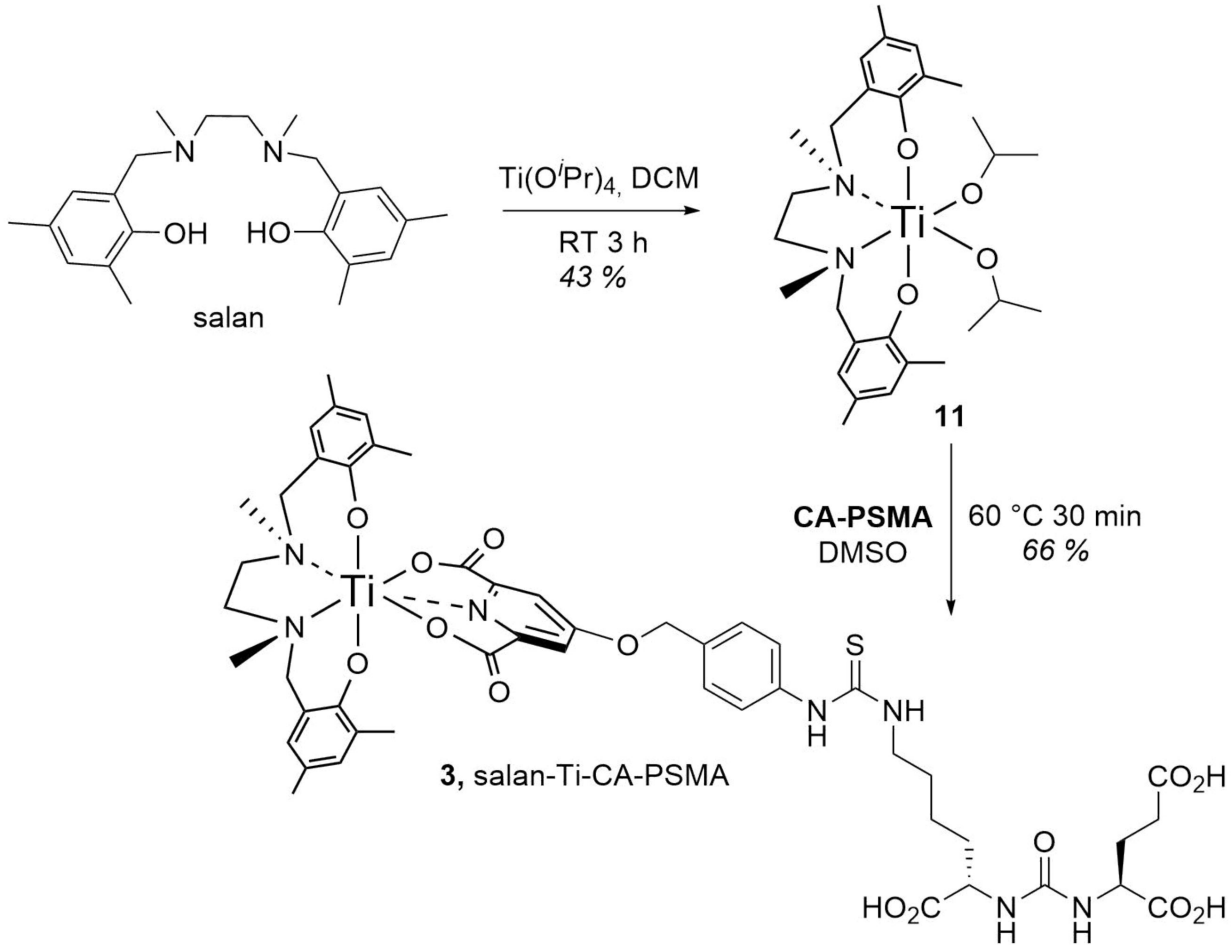
Synthesis of salan-Ti-CA-PSMA (**3**).

The (salan)Ti(O^i^Pr)_2_ complex (**11**) synthesized as described by Chmura et al. [22] was chosen as a precursor for the synthesis of the target compound (**3**) (Scheme 3). In the crowded steric environment imposed by salan, the reaction of (**11**) with (**10**) required elevated temperature producing after 30 min the orange-red salan-Ti-CA-PSMA (**3**) in 66% yield. The compound was further used as reference material for the radio-TLC and radio-HPLC identification of [^45^Ti]salan-Ti-CA-PSMA ([^45^Ti]-**3**).

### 2.3. Production of ^45^Ti and Synthesis of [^45^Ti]salan-Ti-CA-PSMA

^45^Ti was produced by the (p,n) nuclear reaction from scandium foil on a 16.5 MeV GE PETtrace cyclotron. The proton energy was degraded with an aluminum foil to approximately 13 MeV [23] in order to decrease the co-production of titanium-44 (T_½_ = 60 y) by the (p, 2n) nuclear reaction [24]. An experimental saturation yield of 731 ± 234 MBq/µA for thin scandium foil (127 µm) and 1429 ± 397 MBq/µA for thick scandium foil (250 µm) was obtained from irradiation for 10–30 min at 15–20 µA. This corresponded to 66 ± 21% and 64 ± 18%, respectively, of the theoretical saturation yield, which was estimated from cross-sections from the EXFOR database and calculated stopping power using SRIM (Stopping and Range of Ions in Matter) software. It was attempted to achieve a larger ratio of produced ^45^Ti to the mass of scandium to facilitate the purification by LLE. A large amount of scandium challenges the purification of ^45^Ti, since the HCl concentration decreases when dissolving scandium due to the formation of ScCl_3_ and H_2_, and the extraction of ^45^Ti is less efficient at HCl concentrations below 11 M. Furthermore, the amount of nonradioactive titanium introduced by the scandium foil could be reduced and thereby increase the molar activity. Hence, the thin scandium foil was preferred and the size of the foil was reduced from 1–0.7 cm^2^ to 0.4 cm^2^, which decreased the yield of the ^45^Ti production due to the increased risk of not hitting the foil with the entire proton beam profile.

^45^Ti was separated from scandium by LLE with guaiacol/anisole 9/1 (*v/v*) either in batch or by using a Zaiput membrane separator, as described previously [15]. The extraction efficiency with different ratios between the aqueous and organic phases is shown in Table 1. The extraction efficiency was improved with the increased organic phase volume. However, for the further purification of the radiotracer, a large volume of organic solvent was challenging. Hence, a ratio of 1:1.33 (aqueous/organic phase) (*v/v*) was preferred. Another caveat is the addition of a small amount (16 % *v/v*) of pyridine to the ^45^Ti-containing guaiacol/anisole mixture. This neutralizes the traces of HCl in the organic phase, which otherwise might interfere with subsequent radiotitanation.

The synthesis of [^45^Ti]salan-Ti-CA-PSMA ([^45^Ti]-**3**) generally followed a previously developed procedure [18], where the radionuclide was consecutively treated with salan and then with CA-PSMA (**10**) dissolved in DMSO. Different conditions were tested including reaction temperature (60 and 80 °C) and time (5–60 min), concentration of salan and CA-PSMA (2–15 mM), and the addition of pyridine.

**Scheme 4:**
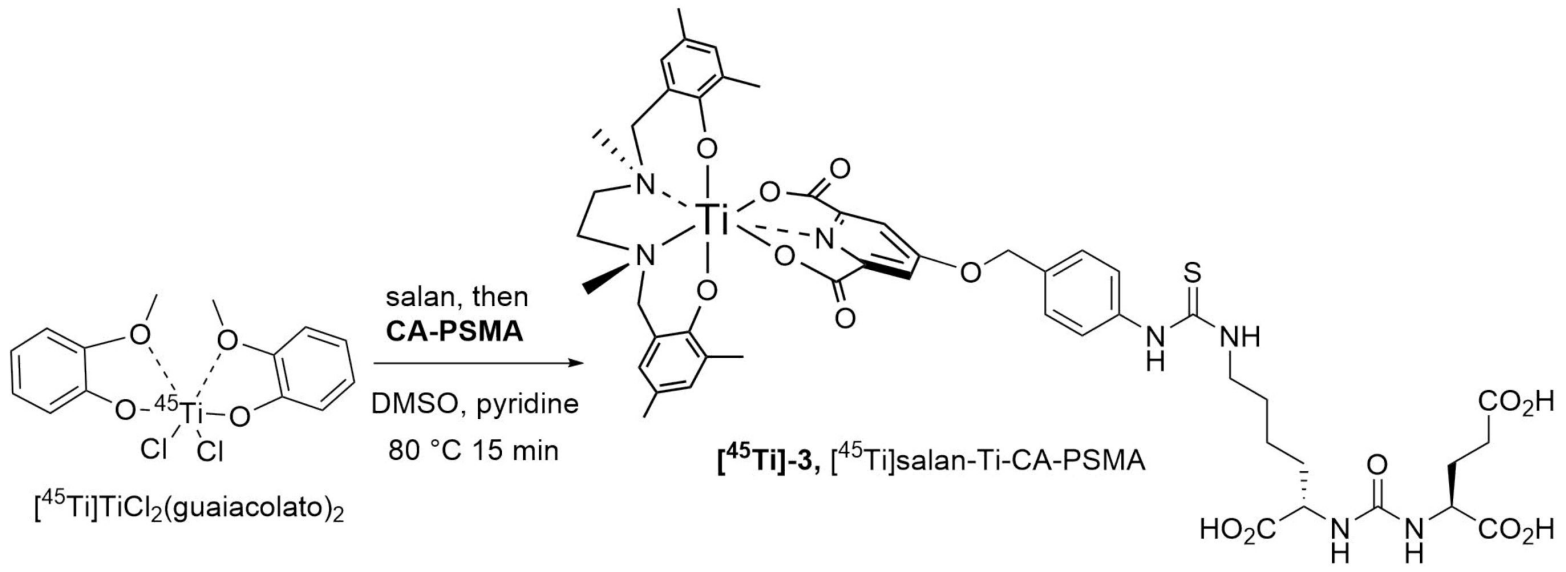
Radiosynthesis of [^45^Ti]salan-Ti-CA-PSMA ([^45^Ti]-**3**).

**Figure 2:**
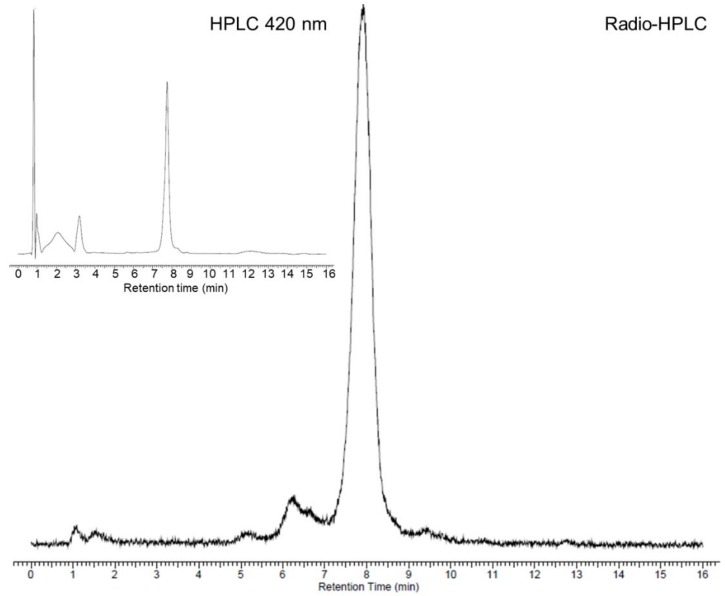
The radio-HPLC chromatogram of the radiolabeling solution after 15 min at 80 °C. The retention time of the peak corresponding to [^45^Ti]salan-Ti-CA-PSMA ([^45^Ti]-**3**) was 7.9 min. Insert: The UV signal at 420 nm of the HPLC chromatogram for the nonradioactive Salan-Ti-CA-PSMA (**3**). The retention time was 7.7 min. The time difference is due to the radioactivity detector being placed after the UV detector.

A radiolabeling yield of 47% was obtained after 60 min at 60 °C with 15 mM of salan and CA-PSMA. No radiolabeling was observed when the concentration of salan and CA-PSMA was decreased to 7.6 mM. When increasing the temperature to 80 °C, a radiolabeling yield of 80%–85% was observed after 15–60 min with 15 mM of salan and CA-PSMA, while no radiolabeling was observed with 2 mM of salan and CA-PSMA. A higher radiolabeling yield could be obtained with 4 mM of salan and CA-PSMA at 80 °C by adding pyridine 1/5 (*v/v*) to the guaiacol/anisole phase, and we found that a reaction time of 15 min was suitable for the formation of [^45^Ti]salan-Ti-CA-PSMA ([^45^Ti]-**3**) (Scheme 4). A total of 86 ± 4 % of ^45^Ti was present as [^45^Ti]salan-Ti-CA-PSMA, which was quantified and identified by radio-HPLC/HPLC with the nonradioactive salan-Ti-CA-PSMA (**3**) as reference material (Figure 2).

Since the radiolabeling was performed in a relatively large volume (6 mL) of high boiling point solvents, an efficient solvent change method was sought after. To that end, the absorption on the silica, alumina N, C18, and QMA (CO_3_^2−^) cartridges was tested first. However, the [^45^Ti]salan-Ti-CA-PSMA ([^45^Ti]-**3**) had either low affinity for the cartridge (C18 and QMA (CO_3_^2−^)) or was difficult to elute afterwards (silica and alumina N). The preparative HPLC (C18 column, eluents: 0.1% formic acid in Milli-Q water (A) and ACN (B)) proved to be the method of choice for the purification of [^45^Ti]salan-Ti-CA-PSMA. The fractions collected from the preparative HPLC were analyzed by analytical radio-HPLC/HPLC in order to identify the fractions containing the product by comparing the retention time to the one for the nonradioactive salan-Ti-CA-PSMA (**3**) reference material. The fractions containing the product were collected and concentrated on a C18 cartridge and eluted in EtOH/H_2_O. The final formulation of [^45^Ti]salan-Ti-CA-PSMA was prepared in phosphate-buffered saline (PBS)/EtOH 9/1 (*v/v*) at pH 7.5. A radiochemical yield (RCY) of 13.0 ± 5.6% decay corrected (d.c.) and 5.1 ± 2.3% non-decay corrected (n.d.c.) was obtained after purification and formulation. The relatively low RCY was partially due to the LLE step, where 25%–40% of the total ^45^Ti was not extracted into the organic phase, and it was partially due to the preparative HPLC, where only 20%–50% of the purified [^45^Ti]salan-Ti-CA-PSMA had a radiochemical purity (RCP) above 95%.

### 2.4. Analyses and Stability Study of [^45^Ti]salan-Ti-CA-PSMA

The RCP of the final product in PBS buffer was 96 ± 3% (n = 7). The radionuclidic purity of the product was determined by gamma spectroscopy using a germanium detector and was found to be 100% as only background radiation and the three most abundant gamma lines of ^45^Ti (511.0, 719.6, and 1408.1 keV) [25] were detected. The molar activity of the product was 110 ± 59 GBq/µmol (n = 7) according to the amount of nonradioactive salan-Ti-CA-PSMA (**3**) in the final formulation of [^45^Ti]salan-Ti-CA-PSMA ([^45^Ti]-**3**) calculated by HPLC. The molar activity was also calculated from the amount of titanium in the product found by inductively coupled plasma optical emission spectrometry (ICP-OES) and was calculated to be 107 GBq/µmol. The nonradioactive titanium content was expected to arise mainly from impurities from the scandium foil; however, the molar activity was anticipated to be sufficient for the animal studies. The amount of other selected metals was also measured by ICP-OES. The amount of scandium was <8.3 µg, the amount of zinc was <5.0 µg, and the amount of iron was 2.0 ± 2.1 µg as found by ICP-OES.

The octanol/water partition coefficient (log P) of [^45^Ti]salan-Ti-CA-PSMA ([^45^Ti]-**3**) was measured at 1.7 in PBS buffer at pH = 7.5.

**Figure 3:**
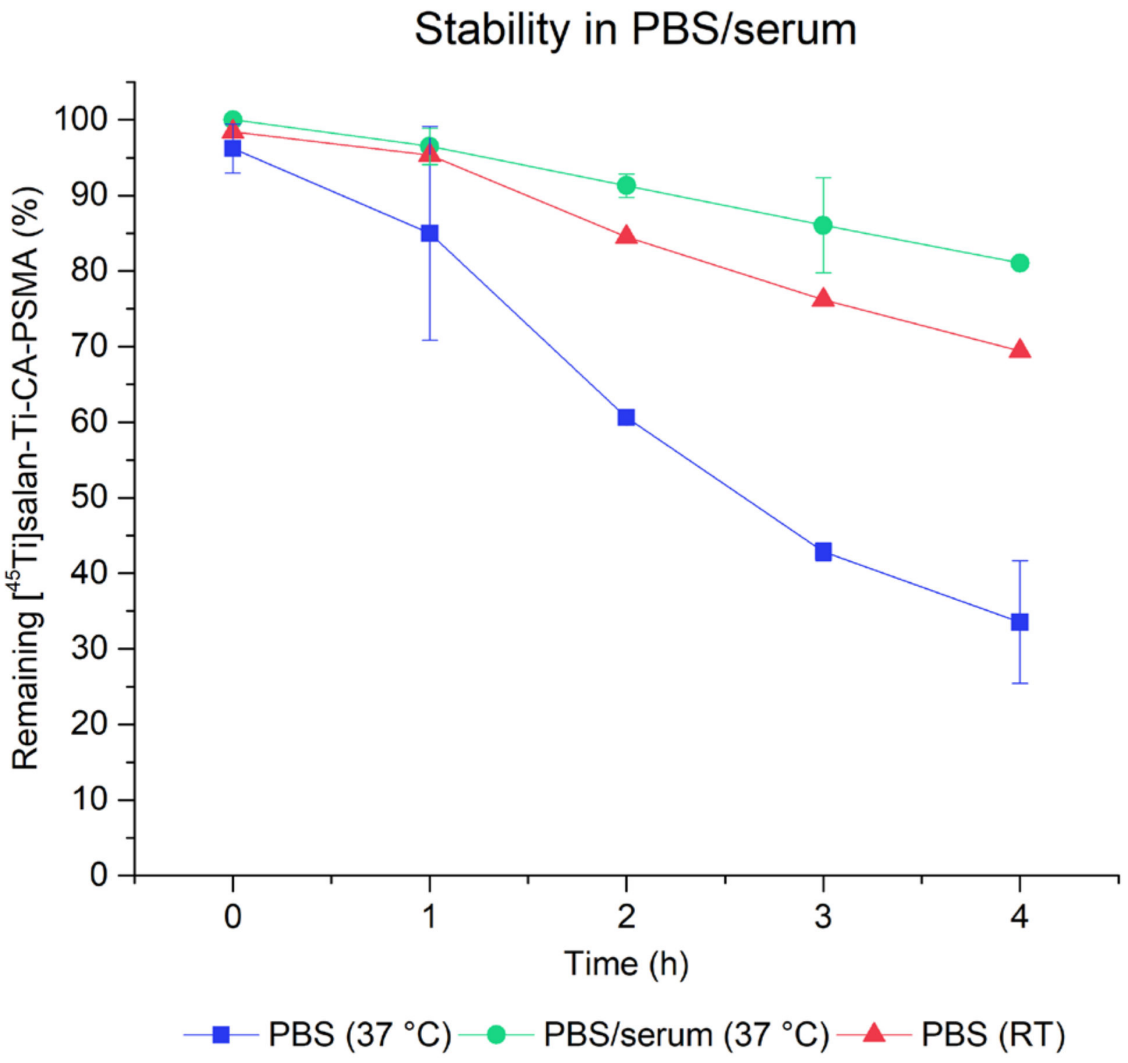
The stability of [^45^Ti]salan-Ti-CA-PSMA ([^45^Ti]-**3**) in PBS buffer at 37 °C (**blue squares**) and at room temperature (**red triangles**) and in PBS/mouse serum 1/1 (*v/v*) at 37 °C (**green circles**). The [^45^Ti]salan-Ti-CA-PSMA was quantified by radio-TLC using nonradioactive salan-Ti-CA-PSMA (**3**) as reference material.

The stability of [^45^Ti]salan-Ti-CA-PSMA in PBS buffer and in mouse serum/PBS buffer 1/1 (*v/v*) at 37 °C and in PBS buffer at room temperature was studied over 4 h by radio-TLC (Figure 3). The percentage of [^45^Ti]salan-Ti-CA-PSMA compared to other [^45^Ti]Ti-compounds was gradually decreasing over time both in PBS buffer and in mouse serum to 33% in PBS buffer and to 80% in mouse serum/PBS buffer 1/1 (*v/v*) after 4 h at 37 °C. The decomposition rate of [^45^Ti]salan-Ti-CA-PSMA in PBS buffer was appreciably slower at room temperature with 69% [^45^Ti]salan-Ti-CA-PSMA remaining intact after 4 h. The radio-TLC showed that the ^45^Ti-containing decomposition products remained at the baseline, suggesting titanium de-chelation and hydrolysis.

### 2.5. In Vivo Study in Mice

**Figure 4:**
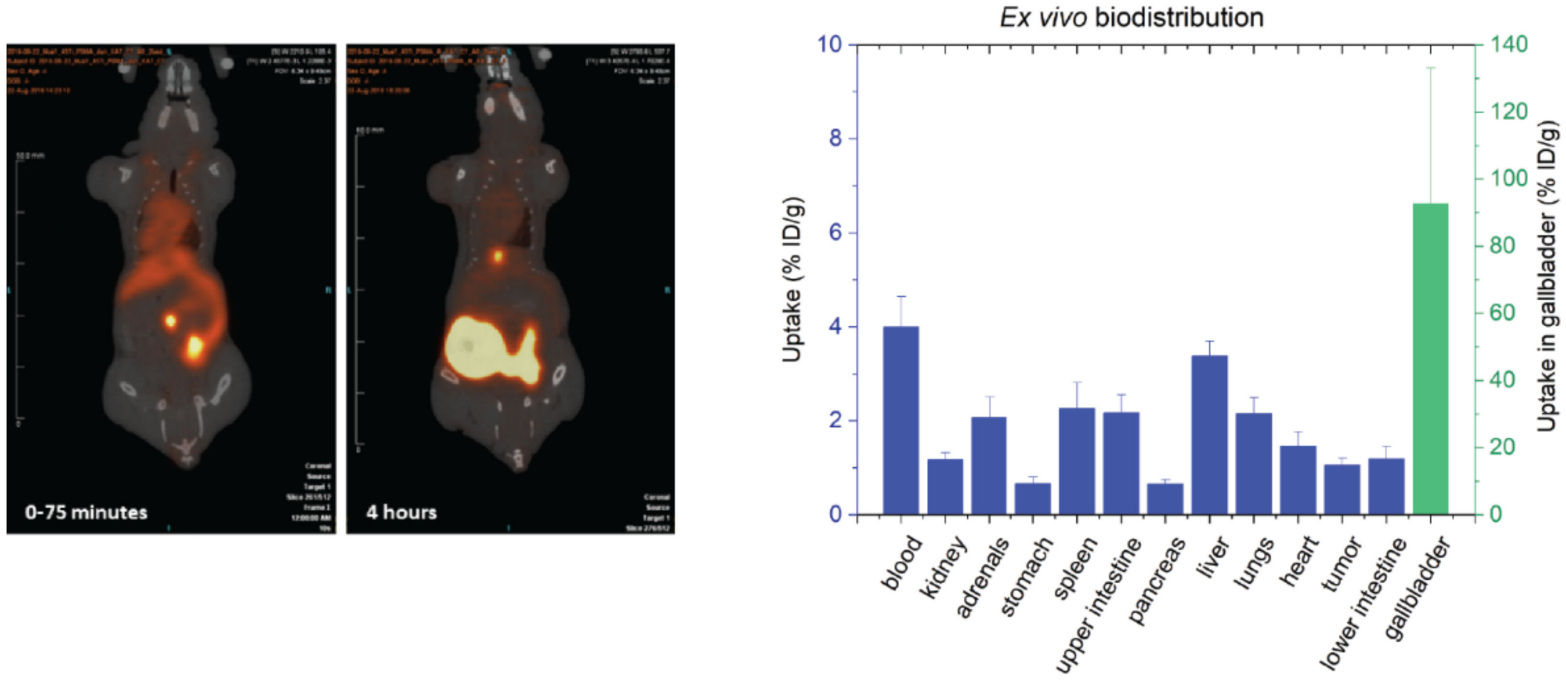
(**left**) PET/CT images (coronal plane) of one of the tumor-bearing mice (mouse no. 1) receiving [^45^Ti]Salan-Ti-CA-PSMA ([^45^Ti]-**3**). Mouse no. 1 was dynamically scanned 0–75 min (left) and four hours (right) post-injection (p.i.); (**right**) The ex vivo biodistribution 4 h p.i. shown as the uptake of ^45^Ti in the percentage of injected dose per weight of tissue. The values were an average for the four mice, and the error bars represent the standard deviation. The uptake in the gallbladder (green) can be read off the right *y*-axis.

The in vivo distribution of [^45^Ti]salan-Ti-CA-PSMA ([^45^Ti]-**3**) in PSMA-positive tumor-bearing mice was conducted with four animals, which all received the same batch of [^45^Ti]salan-Ti-CA-PSMA. The radiotracer was administered at 4, 5.5, 6, and 7.5 h after end of synthesis (EOS), respectively, with a maximal radiochemical purity of 69% (4 h after EOS). The molar activity was 14–30 GBq/µmol depending on the injection time. The microPET scans (Figure 4) showed a high radioactivity accumulation in the gallbladder and intestine. However, no visible uptake of the radiotracer in the tumor, located at the left shoulder of each mouse, was observed. The ex vivo biodistribution (post-mortem) for all four mice was analyzed by measuring the injected dose per gram tissue (% ID/g) of the different organs analyzed (Figure 4). A low amount of radioactivity (1.1 ± 0.1% ID/g) was observed in the tumor tissue; however, this amount was not notably higher than that for the other organs, and it may arise from the blood content. A significant amount of radioactivity per weight of organ was found in the blood (4.0 ± 0.6% ID/g) and in the liver (3.4 ± 0.3% ID/g), while the highest accumulation per weight of organ was observed in the gallbladder (92.7 ± 40.5% ID/g). The gallbladder of the mouse no. 1 was not analyzed since the high accumulation of radioactivity in the gallbladder was noticed after the analysis of the microPET scan of this mouse. A relative large variation between the three other mice was observed for this organ. This variation was expected to be due to the low mass of the gallbladder (6–7 mg), which could cause uncertainty on the mass of the organ. The PET images and the ex vivo biodistribution of the four mice indicated that [^45^Ti]salan-Ti-CA-PSMA ([^45^Ti]-**3**) was metabolized and/or excreted through the hepatobiliary system.

## 3. Discussion

The design of the presented PSMA ligand was guided by two major considerations. Given the hydrolytic stability observed in previous studies [18], we based our chelator design on the salan-Ti-dipic molecular scaffold. The functionalization with the Glu-urea-Lys residue required an exchange of dipic for a structurally related chelidamic acid ester (**4**), which underwent smooth *O*-alkylation with the benzylic isothiocyanate (**5**) and consecutive room temperature coupling with the pharmacophore residue Glu-urea-Lys (**7**). The order of deprotection was important requiring the saponification to (**9**) in order to prevent significant loss of yield, followed by the removal of three ^t^Bu groups to give CA-PSMA (**10**). The complexation with salan of both nonradioactive and ^45^Ti was almost immediate at room temperature, but the attachment of CA-PSMA required forcing conditions, which was likely due to the crowded steric environment imposed by the octahedrally coordinated salan. The LLE of ^45^Ti proved to be a reliable way to recover and purify the radionuclide from 12 M HCl containing a significant amount of Sc. The extraction was found to be scalable and could be performed manually at low levels of activity, or via the syringe pump-driven membrane separator [15]. The latter could be remotely controlled and implemented as a part of an automated radiosynthesis module.

For tracers relying on tight active site binding such as PSMA, the geometrical requirements of the chelator and the length of the linker may play the deciding role. The molecular docking studies we performed on (**2**) clearly indicated that the P1′ and the P1 residues of (**2**) bind in the same active conformation, as does the co-crystallized ligand PSMA-1007 (Figure 1A). The signature interactions with the Zn^2+^; Arg 210, Asn 257, Tyr 552, and Tyr 700; and Lys 699 of the S1′glutamate recognitions site, as well as with Asn 519 and Arg 534 of the S1 carboxylate recognition site of the protein were well reproduced (Figure 1B). Furthermore, the aromatic linker of (**2**) (Scheme 1, blue) was found to be co-planar to the benzylic linker in the co-crystallized PSMA-1007 (Scheme 1, pink), making the backbones of (**1**) and (**2**) well-aligned. This alignment fidelity was also reflected in the comparable DockScores of those compounds, (−8.9 and −12.7, respectively), predicting (**2**) to be a strong binder (comparing those values with the DockScore −0.7 for a typical non-binding decoy molecule). However, the salan-Ti-CA-PSMA (**3**) structurally differs from (**2**) in the attachment of the chelate salan-Ti-CA. The obvious concern was that the chelate might be too sterically big to fit inside the active site of the enzyme. Using quantum chemistry calculations we estimated that salan-Ti-CA will occupy 780 Å^3^ of space. This volume is significantly larger than that Ga-DOTA occupies in the strong binder Ga-PSMA-617 (560 Å^3^). However, the molecular modeling indicated that the linker is long enough for the chelate to extend beyond the narrow binding channel of the active site and occupy a large funnel-shaped entrance of the enzyme (Figure 1C). The closest neighboring side-chains of the enzyme are situated as far as 5 Å from the chelate, relieving it from any steric clashes and the need for conformational changes in the protein.

One feature shared by many clinically tested PSMA PET tracers is their high hydrophilicity: ^68^Ga-PSMA-11 (logP = −4.3) and ^68^Ga-PSMA-617 (logP = −3.4). This predisposition to aqueous media leads to increased accumulation in the kidney and the bladder and can interfere with the detection of prostate lesions. Therefore, the second design consideration was an attempt to moderate the high hydrophilicity imposed by the Glu-urea-Lys pharmacophore. The previous efforts to increase hydrophobicity to improve tumor uptake and internalization focused on placing the hydrophobic residues within the backbone of the inhibitor [26]. This affected binding often in an unpredictable way. To avoid this unpredictability of modifying the linker moiety, we limited ourselves to the non-binding chelate functionality and retained the four methyl groups the salan ligand provided. The measured logP = 1.7 puts salan-Ti-CA-PSMA (**3**) in the category of highly hydrophobic PSMA agents, yet still within the general drug-like Lipinski logP range of values (−1 to +5). Overall, the salan-Ti-CA-PSMA structure was found to be sterically and electronically compatible with the active site of GCII and judged to be an appropriate candidate for in-vivo testing.

The decrease in the concentration of [^45^Ti]salan-Ti-CA-PSMA ([^45^Ti]-**3**) in PBS/serum at 37 °C (approximately 20% loss in 4 h) was a sign of the limited stability of the synthesized compound in vitro (Figure 3). This was later confirmed by the PET and the ex vivo studies. The disappointing uptake of [^45^Ti]salan-Ti-CA-PSMA in the tumor parallels that observed for salan-Ti-dipic we reported earlier [18]. In both cases, the uptake of the radiolabeled material monitored by PET was heavily dominated by the liver and gallbladder with no visible accumulation in the tumor (Figure 4, left). The comparison of the *ex-vivo* biodistribution between the two ^45^Ti-labeled compounds showed that tumor uptake rose from 0.18% ID/g for [^45^Ti]salan-Ti-dipic to 1.1% ID/g for [^45^Ti]salan-Ti-CA-PSMA (Figure 4, right). The similar PET and ex vivo biodistribution profile for the two compounds, which share essentially the same chelate functionality, strongly suggests that the chelate is the culprit of the in vivo instability and hepatobiliary excretion we observed. The competitive binding hypothesis can be used to rationalize the observed behavior. Citrate is known to be a potent molecular binder for Ti(IV) [27] present in blood in approximately 100 µM concentrations [28]. The competitive substitution of the bidentate dipic/CA by the citrate would be close to thermoneutral and driven by the µM concentration of citrate versus sub-nM amounts of ^45^Ti-labeled material. The Tinoco group has recently reported that for highly hydrolytically stable Ti(deferasirox)_2_, the citrate binding facilitates the transmetallation of Ti(IV) by labile Fe(III), which is present inside the cytosol [29]. A similar process is likely to occur here. After the citrate substitution, [^45^Ti]salan-Ti-CA-PSMA ([^45^Ti]-**3**) loses its CA-PSMA (**10**) moiety converting into hydrophobic [^45^Ti]salan-Ti(citrate), which is swept from the bloodstream by hepatocytes. A small portion of [^45^Ti]salan-Ti(citrate) reacts, further releasing [^45^Ti]Ti(citrate)_2_^2−^, which is picked up by transferrin [28] and remains in the blood. In the hepatocytes, [^45^Ti]salan-Ti(citrate) loses its radiotitanium by Fe(III) transmetallation in the cytosol. The hypothesis presented above suggests a strategy for improving the in vivo stability of the titanium chelate. Finding a stronger enthalpic binder for titanium would be an obvious chemical modification. A more practical and perhaps more productive solution is to design a unimolecular chelator by tethering the salan and the CA functionality. This would make the intermolecular citrate substitution thermodynamically non-competitive, effectively blocking the transmetallation of the chelated titanium.

## 4. Materials and Methods

### 4.1. Synthesis of CA-PSMA (**10**) and salan-Ti-CA-PSMA (**3**)

#### 4.1.1. General

All commercially available materials were used as received without further purification. The materials were purchased from Sigma Aldrich (Schnelldorf, Germany), except for glutamic acid di-*t*-butyl ester hydrochloride (98%), O^t^Bu-Lys-Cbz · HCl (97%), *p*-Tolyl isothiocyanate (97%), carbon tetrachloride (99%), chelidamic acid monohydrate (95%), and lithium hydroxide (anhydrous, 95%), which were purchased from abcr GmbH (Karlsruhe, Germany). Anhydrous ethanol from Fluka, methanol (LiChroSolv), DMSO (99%), and TLC plates (silica gel and silica gel 60 RP-18, F_254_) were purchased from Merck (Darmstadt, Germany), and heptane (99.7%), *n*-hexane (97%), chloroform (99.2%), dichloromethane (100%), ethyl acetate (99.9%), acetonitrile (99.9% gradient grade for liquid chromatography), and toluene (HiPer Chromanorm) from VWR were used. Various cartridges (alumina N, C18 plus, QMA, silica plus) were purchased from Waters (Milford, MA, USA) and from ABX (Radeberg, Germany) (QMA preconditioned with CO_3_^2−^).

NMR spectra were recorded with an Agilent 400 MR spectrometer (Santa Clara, CA, USA) operating at 400.445 MHz (^1^H). For analytical HPLC, a Hitachi Chromaster (Tokyo, Japan) equipped with a Carroll and Ramsey 105-S radio-detector and a Hitachi 5430 double diode array detector with a Phenomenex Luna 3 μ C18 (2) column was applied. The UV-signal at 270 and 420 nm was recorded. The eluents used were 0.1% TFA in Milli-Q water (A) and 0.1% TFA in ACN (B). The following gradient was used with a flow rate of 0.5 mL/min: 0–0.1 min: 0% B; 0.1–1 min: 0%–45% B; 1–7 min: 45%–55% B; 7–9 min: 55%–60% B; 9–10 min: 60%–100% B, 10–12 min: 100% B; 12–14: 100%–0% B; 14–16 min: 0% B.

A Shimadzu LC-MS system (Tokyo, Japan) consisting of a CBM-20A communications bus module, a SPD-20A UV-vis detector, a FRC-10A fraction collector, a LC-20AP preparative liquid chromatograph, a FCV-200AL Prep Quaternary Valve, and an LC-MS-2020 was applied for preparative HPLC and Mass spectrometry (MS). A Shim-pack GIST 5 µm C18, 20 × 250mm column was used. The eluents were 0.1% formic acid in Milli-Q water (A) and ACN (B). A flow rate of 15 mL/min and the following gradient were applied: 0–5 min: 0% B; 5–25 min: 0%–60 % B; 25–34 min: 60%–70% B, 34–35 min: 70%–90% B; 35–39 min: 90% B; 39–42 min: 90%–60% B. Fractions of 3 mL were collected automatically with the fraction collector.

#### 4.1.2. Synthesis of diethyl-4-hydroxypyridine-2,6-dicarboxylate (**4**)

Compound **4** was synthesized as described previously [19]. First, 12.6 mL (173 mmol) thionyl chloride was added to 50 mL of anhydrous ethanol in a round-bottom flask under argon flow cooled in an ice bath. Then, 5.03 g (27 mmol) chelidamic acid was added in small portions, and the mixture was stirred for 5 min at 0 °C. The mixture was stirred for 20 h at room temperature, followed by 2 h at reflux. The solvent was removed under reduced pressure. The flask with the crude product was placed in an ice bath, and 50 mL water was added. The mixture was neutralized with 5 mL of 10% aqueous Na_2_CO_3_ and 5 mL of 50% aqueous ethanol. The precipitate was filtered and dried under reduced pressure. The product was obtained as a white solid (5.49 g (85%)). ^1^H NMR (400 MHz, ACN-d_3_) δ 7.65 (s, 2H), 4.39 (q, *J* = 7.1 Hz, 4H), 1.37 (t, *J* = 7.1 Hz, 6H).

#### 4.1.3. Synthesis of 1-(bromomethyl)-4-isothiocyanatobenzene (**5**)

Compound **5** was synthesized as described previously [20]. First, 2.6 g (17.5 mmol) of *p*-Tolyl isothiocyanate was dissolved in 40 mL of CCl_4_ in a round-bottom flask. Then, 3.2 g (18 mmol) *N*-bromosuccinimide was added, followed by a small amount of benzoyl peroxide. The mixture was stirred at reflux for 8 h and then filtered when cooled down to room temperature. The solvent was evaporated, and the crude product was recrystallized in methanol. The product was obtained as pale yellow needle-like crystals (1.9 g (48%)). ^1^H NMR (400 MHz, DMSO-d_6_) δ 7.52 (d, *J* = 8.6 Hz, 2H), 7.42 (d, *J* = 8.6 Hz, 2H), 4.72 (s, 2H). ^13^C NMR (100 MHz, acetone-d_6_) δ 141.1, 141.1, 139.0, 131.6, 126.9, 33.2.

#### 4.1.4. Synthesis of diethyl 4-((4-isothiocyanatobenzyl)oxy)pyridine-2,6-dicarboxylate (**6**)

All starting materials and reagents were dried under vacuum. First, 1.0 g (4.2 mmol, 1 equiv.) of compound **4** was dissolved in 70 mL of dry *N,N*-dimethylformamide (DMF) in a round-bottom flask. Then, 2.7 g (8.3 mmol, 2 equiv.) of Cs_2_CO_3_ was added. The mixture was stirred for 30 min at room temperature. Then, 1.42 g (6.2 mmol, 1.5 equiv.) of compound **5** was dissolved in 6 mL of dry DMF and added dropwise. The mixture was stirred for 2 h at room temperature. The reaction mixture was filtered and DMF was removed. The crude product was dissolved in DCM, filtered, and dried. The product was isolated as a white solid (0.85 g (53%)) by column chromatography with chloroform/ethyl acetate 9/1 (*v/v*). ^1^H NMR (400 MHz, DMSO-d6) δ 7.81 (s, 2H), 7.57 (d, *J* = 8.6 Hz, 2H), 7.49 (d, *J* = 8.6 Hz, 2H), 5.39 (s, 2H), 4.37 (q, *J* = 7.1 Hz, 4H), 1.33 (t, *J* = 7.1 Hz, 6H). ^13^C NMR (100 MHz, DMSO-d6) δ 166.0, 164.1, 149.7, 135.5, 133.6, 129.8, 129.3, 126.2, 114.4, 69.3, 61.7, 14.1.

#### 4.1.5. Synthesis of (*S*)-di-*tert*-butyl 2-(3-((*S*)-6-amino-1-*tert*-butoxy-1-oxohexane-2-yl)ureido) pen-tanedioate (**7**)

The method described by Maresca et al. was applied for the synthesis [21]. First, 1.24 g (4.2 mmol) of triphosgene was dissolved in 20 mL of dry DCM at 0 °C. Then, 4.22 g (11 mmol) of O^t^Bu-Lys-Cbz · HCl and 4.3 mL (24 mmol) of *N,N*-diisopropylethylamine (DIPEA) in 20 mL dry DCM was added dropwise over 1 h. A solution of 3.35 g (11 mmol) OtBu_2_-Glu · HCl and 4.3 mL (24 mmol) DIPEA in 20 mL of dry DCM was added in one portion, and the mixture was stirred for 1 h. The solvent was evaporated, and the crude product was dissolved in 60 mL of ethyl acetate and washed twice with 80 mL of 2 M NaHSO_4_. The organic phase was washed with brine and dried over Na_2_SO_4_. Na_2_SO_4_ was removed by filtration, and the crude product was dried. The product (2.47 g (36%)) was obtained by column chromatography with hexane/ethyl acetate 2/1 (*v/v*) as eluent. (The product was also synthesized by first adding OtBu_2_-Glu · HCl dropwise to the triphosgene solution and then adding O^t^Bu-Lys-Cbz · HCl in one portion afterwards.) ^1^H NMR (400 MHz, CDCl_3_) δ 7.32 (m, 5H), 5.24–5.12 (m, 3H), 5.09 (d, *J* = 5.8 Hz, 2H), 4.37–4.28 (m, 2H), 3.22–3.12 (m, 2H), 2.36–2.20 (m, 2H), 2.10–2.00 (m, 1H), 1.88–1.71 (m, 2H), 1.66–1.56 (m, 1H), 1.54–1.47 (m, 2H), 1.46–1.41 (m, 27H), 1.40–1.30 (m, 2H). ^13^C NMR (100 MHz, CDCl_3_) δ 172.6, 172.5, 172.4, 157.0, 156.7, 136.8, 128.6, 128.2, 128.2, 82.3, 81.9, 80.7, 66.7, 53.4, 53.1, 40.8, 32.7, 31.7, 29.5, 28.5, 28.2, 28.1, 22.4.

For Cbz deprotection, 0.63 g (1.0 mmol) of ammonium formate was suspended in 10 mL of EtOH. Then, 0.62 g (1 mmol) of the Cbz-protected compound from the previous step was dissolved in 10 mL of ethanol and added to the suspension. Then, 67 mg of 10% Pd-C was added, and the mixture was stirred at room temperature overnight. The mixture was filtered through a celite pad, and the solvent was removed under reduced pressure. The remaining ammonium formate was removed by dissolving the crude product in 50 mL of DCM and washing with 50 mL of 1 M Na_2_CO_3_ aqueous solution. The organic phase was washed with brine and dried over Na_2_SO_4_. The solvent was removed under reduced pressure, and the product was obtained (0.41 g (84%)). ^1^H NMR (400 MHz, CDCl_3_) δ 6.41–6.20 (m, 2H), 4.36–4.26 (m, 2H), 3.07–2.92 (m, 2H), 2.34–2.89 (m, 2H), 2.12–2.01 (m, 1H), 1.89–1.61 (m, 5H), 1.55–1.38 (m, 28H), 1.28–1.23 (m, 1H). ^13^C NMR (100 MHz, DMSO-d_6_) δ 172.3, 172.0, 171.5, 157.3, 80.5, 80.3, 79.8, 53.1, 52.2, 38.6, 31.3, 30.9, 27.8, 27.7, 27.6, 22.1.

#### 4.1.6. Synthesis of diethyl 4-((4-(3-((*S*)-6-(*tert*-butoxy)-5-(3-((*S*)-1,5-di-*tert*-butoxy-1,5-dioxopentan-2-yl)ureido)-6-oxohexyl)thioureido)benzyl)oxy)pyridine-2,6-dicarboxylate (**8**)

First, 0.75 g (19 mmol) of compound **6** was dissolved in 20 mL of dry DMF in a round-bottom flask. Then, 0.95 g (19 mmol) of compound **7** was dissolved in 20 mL of dry DMF and added dropwise to the flask. The mixture was stirred at room temperature for 30 min. The solvent was evaporated, and the product was dried under reduced pressure. The product was purified by column chromatography with hexane/ethyl acetate 1/2 (*v/v*) and 1.36 g (82%) of compound **8** was obtained. ^1^H NMR (400 MHz, DMSO-d_6_) δ 7.81 (s, 2H), 7.44 (m, 4H), 6.28 (m, 2H), 5.31 (s, 2H), 4.38 (q, *J* = 7.1 Hz, 4H), 4.07–3.96 (m, 2H), 3.46 (m, 2H), 2.29–2.15 (m, 2H), 1.91–1.82 (m, 1H), 1.71–1.50 (m, 5H), 1.40–1.37 (m, 28H), 1.34 (t, *J* = 7.1Hz, 6H), 1.36–1.29 (m, 1H). ^13^C NMR (100 MHz, DMSO-d_6_) δ 180.3, 172.2, 171.9, 171.4, 166.1, 164.1, 157.1, 149.7, 139.5, 130.8, 128.6, 122.7, 114.3, 80.6, 80.3, 79.7, 70.0, 61.6, 53.1, 52.1, 43.6, 31.8, 28.1, 27.7, 27.6, 22.5, 14.1.

#### 4.1.7. Synthesis of 4-((4-(3-((*S*)-6-(*tert*-butoxy)-5-(3-((*S*)-1,5-di-*tert*-butoxy-1,5-dioxopentan-2-yl)ureido)-6-oxohexyl)thioureido)benzyl)oxy)pyridine-2,6-dicarboxylic acid (**9**)

First, 0.25 g (0.29 mmol) of compound **8** was dissolved in 7 mL of THF and 14 mg (0.58 mmol, 2.2 equiv.) LiOH in 3 mL water was added. The mixture was stirred for 3.5 h at room temperature. The solvent was removed under reduced pressure, and the product was obtained as the lithium salt (0.22 g (91%)) without further purification. ^1^H NMR (400 MHz, DMSO-d_6_) δ 7.60 (d, *J* = 8.0 Hz, 2H), 7.55 (s, 2H), 7.36 (d, *J* = 8.4 Hz 2H), 6.40 (m, 2H), 5.20 (s, 2H), 4.00 (m, 2H), 3.46 (m, 2H), 2.30–2.14 (m, 2H), 1.90–1.81 (m, 1H), 1.71–1.50 (m, 5H), 1.40–1.36 (m, 27H), 1.36–1.27 (m, 2H). ^13^C NMR (100 MHz, DMSO-d_6_) δ 180.4, 172.3, 171.9, 171.5, 167.6, 166.2, 157.2, 156.4, 140.0, 128.0, 122.5, 110.0, 80.6, 80.3, 79.7, 69.2, 53.2, 52.2, 43.5, 31.8, 30.9, 30.4, 28.2, 27.7, 27.7, 22.7.

#### 4.1.8. Synthesis of 4-((4-(3-((*S*)-5-carboxy-5-(3-((*S*)-1,3-dicarboxypropyl)ureido)pentyl)thioureido)-benzyl)oxy)pyridine-2,6-dicarboxylic acid (CA-PSMA) (**10**)

First, 0.20 g (0.24 mmol) of compound **9** was mixed with 6 mL of TFA/DCM 1/1 (*v/v*) and stirred at room temperature for 2.5 h. The product was precipitated in 30 mL of cold diethyl ether and washed three times with cold diethyl ether. The product was dried and the product was obtained as a white solid (0.13 g (84%)). ^1^H NMR (400 MHz, DMSO-d_6_) δ 7.74 (s, 2H), 7.48 (d, *J* = 8.7 Hz, 2H), 7.41 (d, *J* = 8.5 Hz, 2H), 6.36 (m, 2H), 5.32 (s, 2H), 4.08 (m, 2H), 3.44 (m, 2H), 2.29–2.19 (m, 2H), 1.96–1.87 (m, 1H), 1.76–1.64 (m, 2H), 1.60–1.50 (m, 3H), 1.38–1.28 (m, 2H). ^13^C NMR (100 MHz, DMSO-d_6_) δ 180.3, 174.5, 174.2, 173.7, 170.1, 167.4, 164.5, 157.3, 150.3, 139.4, 128.6, 122.6, 113.2, 70.2, 52.2, 51.6, 43.7, 31.9, 29.9, 28.2, 27.5, 22.8.

#### 4.1.9. Synthesis of (6,6′-((ethane-1,2-diylbis(methylazanediyl))bis(methylene))bis(2,4-dimethyl-phenol))-diisopropoxide titanium(IV) (salan)Ti(O^i^Pr)_2_ (**11**)

(salan)Ti(O*^i^*Pr)_2_ (**11**) was synthesized as described by Chmura et al. [22]. First, 0.44 g (1.5 mmol) of titanium isopropoxide was transferred to a round-bottom flask under argon flow and dissolved in 12 mL of dry DCM. Then, 0.54 g (1.5 mmol) of salan was dissolved in 3 mL of dry DCM and added to the round-bottom flask. The mixture was stirred for 3 h at room temperature. The solvent was evaporated, and the crude product was recrystallized in heptane. The product was obtained as yellow crystals (0.33 g (43 %)). ^1^H NMR (400 MHz, CDCl_3_) δ 6.89 (d, *J* = 1.5 Hz, 2H), 6.61 (d, *J* = 1.5 Hz, 2 H), 5.17 (sept., *J* = 6.1 Hz, 2H), 4.69 (d, *J* = 13.3 Hz, 2H), 3.05 (d, *J* = 13.3 Hz, 2H), 3.04 (d, *J* = 9.4 Hz, 2H), 2.41 (s, 6H), 2.24 (s, 6H), 2.22 (s, 6H), 1.73 (d, *J* = 9.5 Hz, 2H), 1.28 (d, *J* = 6.1 Hz, 6H), 1.20 (d, *J* = 6.1 Hz, 6H).

#### 4.1.10. Formation of salan-Ti-CA-PSMA (**3**)

First, 20 mg (0.04 mmol) (salan)Ti(^i^OPr)_2_ (**11**) and 25 mg (0.04 mmol) of CA-PSMA (**10**) were dissolved in 1.5 mL of DMSO. The reaction mixture was stirred for 30 min at 60 °C. The product was precipitated in diethyl ether and washed twice with diethyl ether. Then, 20 mg of the crude product was purified by preparative TLC (C18 plates) using ACN/H_2_O 1/1 (*v/v*) as eluent. The product was collected from the plate (R_f_: 0.5-0.6), eluted from the C18 silica with acetone, filtered, and dried (13 mg (66 %)). ^1^H NMR (400 MHz, DMSO-d_6_) δ 7.69 (s, 2H), 7.45 (m, 4H), 6.78 (s, 2H), 6.76 (s, 2H), 6.33 (m, 2H), 5.47 (d, *J* = 11.7 Hz, 1H), 5.42 (d, *J* = 11.7 Hz, 1H), 4.92 (d, *J* = 13.7 Hz, 2H), 4.08 (m, 2H), 3.44 (m, 2H), 3.32 (d, *J* = 13.8 Hz, 2H), 3.12 (d, *J* = 8.8 Hz, 2H), 2.6 (s, 6H), 2.3 (d, *J* = 8.9 Hz, 2H), 2.27–2.20 (m, 2H), 2.15 (s, 6H), 1.95–1.87 (m, 1H), 1.83 (s, 6H), 1.75–1.64 (m, 2H), 1.58–1.49 (m, 3H), 1.35–1.28 (m, 2H). ^13^C NMR (100 MHz, DMSO-d_6_) δ 180.2, 174.6, 174.3, 173.8, 170.6, 167.9, 157.3, 155.5, 150.9, 139.7, 135.5, 130.2, 129.6, 128.9, 127.9, 127.2, 123.8, 122.8, 111.4, 71.4, 53.1, 52.3, 51.7, 45.9, 31.9, 30.0, 28.2, 27.6, 22.8, 20.3, 15.4. MS (*m/z*): [M + H]^+^ calcd for C_49_H_59_N_7_O_14_S, 1050.96; found, 1050.

The radiolabeling conditions were tested, where 2 mL of 0.01 M (0.02 mmol) TiCl_4_ in 12 M HCl was mixed with 2 mL of guaiacol/anisole 9/1 (*v/v*) for 10 min. The mixture was centrifuged, and the two phases were separated with a pipette. Then, 0.4 mL pyridine was added to 1 mL of the organic phase. Then, 4 mg (0.01 mmol) salan in 0.4 mL of DMSO was added, and the mixture was stirred at 80 °C for 5 min. Then, 7.5 mg (0.01 mmol) of CA-PSMA (**10**) in 0.4 mL of DMSO was added, and the reaction mixture was stirred for 30 min. The product was purified using preparative HPLC. The fractions were analyzed by analytical HPLC, the fractions containing the product were collected, and the solvent was evaporated. The product was dried and analyzed by NMR, which gave identical spectra to Salan-Ti-CA-PSMA (**3**) synthesized by the previous method.

### 4.2. Production of ^45^Ti and Synthesis of [^45^Ti]salan-Ti-CA-PSMA ([^45^Ti]-**3**)

#### 4.2.1. General

The scandium foil (250 µm and 127 µm, 99.9% pure, rare earth analysis) was purchased from Alfa Aesar, and the aluminum foil (500 µm, 99.9%) was purchased from VWR. Hydrochloric acid (37%, trace metal basis) was purchased from Honeywell, and guaiacol and anisole (99%) from Sigma Aldrich were used for the LLE of ^45^Ti. In addition, 15 mL plastic centrifuge tubes with screw caps (SuperClear) were purchased from VWR, and the membrane separator was purchased from Zaiput Flow Technologies. Perfluoroalkoxy alkane (PFA) diaphragms from McMaster Carr., Pall polytetrafluoroethylene (PTFE) membranes (0.2 µm pore size, 139 µm thickness, PTFE, polypropylene (PP) support), PFA tubing (1/16” OD, 0.03” ID and 1/8” OD, 1/16” ID) from Idex Health and Science, and static mixers (PTFE, 10 element, 1.7 cm length, placed inside a piece of 1/8” OD, 1/16” ID PFA tubing) from Stamixco were used. Pyridine (anhydrous, 99.8%) and ICP standards (multi-element standard solution 1 for ICP (TraceCert in 10% HNO_3_), titanium standard for ICP (1000 mg/L, TraceCert in 2% HNO_3_), and scandium standard for ICP (1000 mg/L, TraceCert in 5% HNO_3_)) from Sigma Aldrich were purchased.

^45^Ti was produced with a GE 16.5 MeV PETtrace cyclotron. A CRC-55tR, CII Capintec, Inc. dose calibrator and a Princeton Gammatech LGC 5 germanium detector were used to measure the radioactivity. For the batch LLE, an IKA ROCKER 3D digital shaker for phase mixing and an Eppendorf 5702 centrifuge for phase separation were applied. For the LLE in flow, KDS 100 Legacy Syringe Pumps were used. A Perkin Elmer Cyclone Plus imager and a Wolf Trimline LED illuminator were applied for radio-TLC. A Thermo Scientific iCAP 6000 Series ICP Optical Emission Spectrometer was used for ICP-OES measurements. For microPET/CT scans, a Siemens INVEON multimodality scanner in a docked mode (Siemens pre-clinical solutions, Knoxville, TN, US) was used.

#### 4.2.2. Production and Purification of ^45^Ti

First, 0.3–2.0 GBq ^45^Ti was produced from 250 µm or 127-µm thick scandium foil by the ^45^Sc(p,n)^45^Ti nuclear reaction using a 16.5 MeV GE PETtrace cyclotron. A 500-µm thick aluminum foil was used to degrade the beam energy to approximately 13 MeV. The mass of the scandium foil used in the target was between 22 and 80 mg and the foil was cut into squares with scissors. The scandium foil was irradiated for 20–30 min at 15–20 µA and was dissolved in 3–6 mL 12 M HCl within 5-10 min. The solution was filtered and centrifuged. ^45^Ti was separated from scandium by LLE with guaiacol/anisole 9/1 (*v/v*) either in flow by the membrane-based separation method or in batch mode. A Zaiput membrane separator with a 2 mil thick PFA diaphragm for pressure control was used for the LLE in flow. The organic and the aqueous phase were loaded in respective syringes and pumped through PFA tubing using two syringe pumps. An organic flow rate of 0.75 mL/min and aqueous flow rate of 0.25 mL/min were applied. The two phases were mixed when entering a polyether ether ketone (PEEK) tee and passed two 10-element static mixers placed in a piece of tubing. Hereafter, steady slug flow was developed throughout 108 cm of PFA tubing, where the two phases were mixed further. Then, the mixed phases entered the separator, where the organic phase permeated a hydrophobic PTFE/PP) membrane with a pore size of 0.2 µm, while the aqueous phase was retained. The organic phase was collected from the permeate outlet and the aqueous phase was collected from the retentate outlet.

For the LLE in batch mode, the two phases were mixed for 10 min using a digital shaker, centrifuged, and separated with a pipette. The ratio between the organic and aqueous phase was 1:1, 1:1.33, or 1:3 (aq:org) with the dissolved, irradiated scandium foil in 12 M HCl as the aqueous phase and guaiacol/anisole 9/1 (*v/v*) as the organic phase.

#### 4.2.3. Formation and Purification of [^45^Ti]salan-Ti-CA-PSMA ([^45^Ti]-**3**)

The ^45^Ti-labeling was performed by mixing 0.5 mL or 2 mL of the organic phase (guaiacol/anisole) containing ^45^Ti (60–130 MBq/mL, molar activity: 0.5–1.1 GBq/µmol) from the LLE with 8 mg (0.02 mmol) of salan dissolved in 0.5 mL of DMSO at 60 °C for 5 min. Then, 15 mg (0.02 mmol) of CA-PSMA (**10**) dissolved in 0.5 mL of DMSO was added to the reaction mixture and stirred at 60 °C for 60 min.

The ^45^Ti-labeling was also performed at 80 °C, where the same amount of organic phase and of salan and CA-PSMA (**10**) were applied. Salan and CA-PSMA (**10**) were each dissolved in 0.5 mL or 2 mL of DMSO. Finally, the labeling was performed with 4 mL of organic phase containing ^45^Ti (190–225 MBq/mL, molar activity: 43–220 GBq/µmol), where 0.8 mL of pyridine and 8 mg (0.02 mmol) of salan dissolved in 0.8 mL DMSO were added and stirred at 80 °C for 5 min. Then, 15 mg (0.02 mmol) of CA-PSMA (**10**) dissolved in 0.8 mL of DMSO was added to the reaction mixture and stirred for 15 min at 80 °C.

The formation of [^45^Ti]Salan-Ti-CA-PSMA ([^45^Ti]-**3**) was followed by radio-HPLC, where the retention time of the radio peak was compared to the UV signal of salan-Ti-CA-PSMA (**3**). The radiolabeling solution was diluted with toluene 1/1 (*v/v*) and loaded on a silica plus or an alumina N cartridge. The cartridge was washed with toluene, and the product was eluted with 20%–50% H_2_O in MeOH or BuOH/H_2_O/AcOH 4/1/1. It was also attempted to trap the product on a C18 plus cartridge (conditioned with 5–10 mL EtOH followed by 5–10 mL water) and elute the product with ACN/H_2_O 6/4 (*v/v*). The radiolabeling solution was also loaded on a QMA cartridge preconditioned with carbonate solution and washed with water. The cartridge was washed with water and DMSO.

The radiolabeling solution was also injected onto a preparative HPLC with a C18 column, and fractions were collected automatically. The fractions were analyzed by measuring the radioactivity with a dose calibrator and evaluating the radiochemical identity by analytical radio-HPLC. The fractions containing the product were collected and diluted with H_2_O 1/1 (*v/v*) before being passed through a C18 plus cartridge (conditioned with 5–10 mL ethanol followed by 5–10 mL H_2_O). The cartridge was washed with H_2_O and [^45^Ti]Salan-Ti-CA-PSMA ([^45^Ti]-**3**) was eluted in 1.5 mL ethanol/H_2_O 9/1 (*v/v*). This solution was dried down to 50–100 µL under argon flow and was diluted with PBS buffer (pH 7.5).

### 4.3. Analyses and Stability Studies of [^45^Ti]salan-Ti-CA-PSMA ([^45^Ti]-**3**)

The RCP of the final product in PBS buffer after the end of synthesis and purification was analyzed by radio-HPLC. The HPLC-peak expected to arise from the [^45^Ti]Salan-Ti-CA-PSMA ([^45^Ti]-**3**) was identified by comparing the retention time to the one of the nonradioactive Salan-Ti-CA-PSMA (**3**). The RCP was calculated by dividing the area of the [^45^Ti]Salan-Ti-CA-PSMA peak with the total peak area.

The radionuclidic purity was determined by gamma spectroscopy. A Germanium detector, which was calibrated with barium-133 and europium-152 sources, was applied. The samples containing approximately 3 MBq of ^45^Ti were measured in a distance of 1 m for 0.3–3 h. ^45^Ti was identified from the three gamma lines with highest intensity (511.0 keV (169.6%), 719.6 keV (0.154%), and 1408.1 keV (0.085%) [25]). The radionuclidic purity was calculated by dividing the activity of ^45^Ti with the total activity of the sample.

The amount of metals (Ti (323.4 nm), Sc (357.2 nm), Fe (239.5, 240.4, 259.8 nm), and Zn (202.5, 206.2, 213.8 nm)) in the final product was analyzed by ICP-OES at the listed wavelengths. Then, 50 µL of the [^45^Ti]Salan-Ti-CA-PSMA ([^45^Ti]-**3**) in the final eluate solution (EtOH/H_2_O 9/1 (*v/v*)) was diluted to 10 mL with 1% HCl. The concentrations of metals were calculated from a standard curve of samples with known concentrations. The molar activity was calculated from the amount of titanium found by ICP-OES or from the concentration of Salan-Ti-CA-PSMA (**3**) calculated from the UV-signal of the product seen on the analytical HPLC from a standard curve.

The octanol/water partition coefficient of [^45^Ti]Salan-Ti-CA-PSMA ([^45^Ti]-**3**) was measured by taking 0.5 mL of the eluate from the C18 cartridge, which was evaporated to dryness. Then, 1 mL of PBS buffer and 1 mL octanol were added to the dried product. The two phases were mixed for 2 min and subsequently separated. The radioactivity of 0.5 mL of both phases was measured with a dose calibrator. The partition coefficient was calculated by dividing the activity in the octanol phase (A_45Ti,octanol_) with the activity in the aqueous phase (A_45Ti,water_), and the log P value was calculated by Equation (1).

(1) $$\log\;P=\log\frac{A_{45_{\mathrm{Ti},\mathrm{octanol}}}}{A_{45_{\mathrm{Ti},\mathrm{water}}}}$$

The stability of [^45^Ti]Salan-Ti-CA-PSMA ([^45^Ti]-**3**) was analyzed in PBS buffer at room temperature and 37 °C and in PBS buffer/mouse serum 1/1 (*v/v*) and PBS/H_2_O 1/1 (*v/v*) at 37 °C. The samples were analyzed by radio-TLC after 0, 1, 2, 3, and 4 h using C18 TLC plates and ACN/H_2_O 7/3 (*v/v*) as eluent. The nonradioactive Salan-Ti-CA-PSMA (**3**) was used as a TLC reference, and the plates were analyzed using a Perkin Elmer Cyclone Plus imager.

### 4.4. Computational Studies

The geometry optimization for the chelate salan-Ti-CA and Ga-DOTA were performed using Turbomole 7.4.1 at the BP/TZVP level, and the molecular volume was calculated using CosmothermX 19.0.1. For molecular docking studies performed using the Schrodinger 2018 suite of programs, the crystal structure from the Protein Data Bank (PDB ID: 5O5T, 1.43Å resolution) was used. All waters of crystallization were removed except for water 1233, which was complexed to the two zinc atoms of the active site. The protein and the ligand were pre-processed using the default settings of the protein and ligand preparation routines. The flexible docking was carried out at standard precision (SP) using Glide and then refined using extra precision (XP) settings.

### 4.5. In Vivo Study in Mice

The in vivo studies in mice were performed at Odense University Hospital. The animal experiment license number was 2016-15-0201-01027.

Four male Balb/c nude mice (Janvier) received an injection with 5 million PC3+ cells at the left shoulder. The tumors were allowed to grow for 24 days. The tumor sizes were between 7 × 6 and 9 × 9.5 mm, and the mice weighed 25–27 g two days before the in vivo study. A Siemens INVEON multimodality scanner (Siemens pre-clinical solutions, Knoxville, TN, US) was applied for the PET/CT scans. A CT scan of 15 min was performed prior to each PET scan. The mice were anesthetized with a mixture of 1.5%–2% isoflurane and 100% oxygen during injection of the radiotracer and during the scans, where the mice were placed feet first in a prone position on a heated PET/CT animal bed. Each mouse received 0.5–1.4 MBq of [^45^Ti]Salan-Ti-CA-PSMA ([^45^Ti]-**3**) in PBS buffer containing 10% EtOH administered intravenously through the tail vein. The first mouse received the [^45^Ti]Salan-Ti-CA-PSMA tracer 4 h EOS, while the fourth mouse received the tracer 7.5 h EOS. The two first mice were scanned by dynamic acquisition from 0–75 min followed by 20 min static scan four hours p.i. CT and PET images were co-registered using a transformation matrix and CT-based attenuation correction was applied to the PET data. The PET data was reconstructed using an OSEM3D/MAP algorithm (matrix 128 × 128, 2 OSEM3D and 18 MAP iterations). The two last mice received a 15 min static scan one hour p.i., followed by a 20 min static scan four hours p.i. After the last scanning, the mice were euthanatized by cervical dislocation and dissected. The organs were collected and weighed separately. The amount of ^45^Ti in the organs was measured with a gamma well counter.

## 5. Conclusions

The development of titanium pharmaceuticals and radiopharmaceuticals so far has been unsuccessful with all drug candidates consistently failing in vivo. Bringing together new methods of ^45^Ti purification, computer-aided design, and synthesis of the new ^45^Ti-containing PSMA ligand, this work takes another step toward creating the first target-specific ^45^Ti PET tracer. The molecular docking of salan-Ti-CA-PSMA (**3**) into the active site of GCPII indicated the possibility of the signature binding of the Glu-urea-Lys pharmacophore without creating prohibitive steric interaction of the protein with the bulky salan-Ti-CA chelator. Although the new compound yielded no visible PET signature in the tumor and poor ex vivo tumor accumulation, the analysis of the data and the literature may suggest the involvement of competitive citrate substitution and Ti transchelation as the pathway for in vivo de-titanation. A chemical elaboration of the structure to create a unimolecular chelator could be a practical way to increase the in vivo stability of future ^45^Ti-containing radiopharmaceuticals.

## Figures and Tables

**Table 1:** The average extraction efficiency of ^45^Ti at different ratios between the aqueous and organic phases. The mass of scandium foil, the HCl concentration after dissolving the scandium foil, the mass of scandium per volume, and the number of experiments are listed.

**Sc Foil (mg)**	**Concentration of HCl after Dissolving Foil (M)**	**Sc/HCl** **(mg/mL)**	**Aq:org Ratio**	**Average Extraction %**	**Number of Experiments**
42–80	10.9–12.0	2.6–18.7	1:3	77.7 ± 10.8	7
22–36	11.4–11.7	7.3–12.0	1:1.33	68.0 ± 6.5	5
31–57	11.2–12.0	2.3–14.3	1:1	59.0 ± 5.8	8
